# Zinco-Magnesium Light

**Published:** 1867-12

**Authors:** B. Wood

**Affiliations:** Albany, N. Y.


### ZINCO-MAGNESIUM LIGHT.

BY B. WOOD, M. D., D. D. S.

It is only within the past ten years that the wonderful illuminating power of the *Magnesium Light* has commanded public notice. M. Bussy, in 1830, obtained the metal in sufficient quantity to determine its properties; (Davy had previously proved its existence,) but it remained merely an object of scientific inquiry, until, by improved processes of preparation, it was produced in sufficient amount to suggest its practical application in the arts, when it at once became invested with general interest. To M. Deville of France, who was so successful in the preparation of aluminum, do we owe, perhaps more than to any one else, the placing of this other still more remarkable metal within the reach of art.

In the *Comptes Rendus* for February 23, 1857, M. M. Deville and Caron gave a detailed paper on the preparation of magnesium, being similar to the processes employed in the reduction of aluminum; and also communicated some information respecting the physical characters of the metal not before determined. Its density is 1.75. It fuses at about the melting point of zinc, at a low red heat, and at a little higher temperature bursts into a dazzling white flame, attended with phenomena observable in the combustion of zinc. Like zinc it is volatile, and at nearly the same temperature. An ounce could be easily distilled at a time.

Shortly after, M. Bunsen of Paris, proposed the employment of the metal in the form of fine wire for illuminating purposes. He found a wire one-hundredth of an inch in diameter to burn at the rate of about three feet in a minute, and give a light equal to seventy-four stearine candles. The light, according to his estimate, is only thirteen times less than actual sunlight. He proposed having the wire wound upon bobbins, and from these paid out to the lamp. Since then, no little ingenuity has been expended in devising and perfecting suitable means and arrangements for utilizing this light; especially for taking photographic views at night, and in caverns and other obscure recesses, for which purpose it is so valuable for its great actinic power; the chief practical difficulty being the rapidity with which the metal is consumed, involving great inconvenience as well as cost.

The *London Photographic News*, in an article urging the claims of the magnesium light over other artificial lights in photogeny, (quoted the *Scientific American* for August 8, 1863,) describes an arrangement then recently devised, wherein "a spool of wire is gradually unwound, the end being pushed horizontally into the flame of a spirit lamp, where it ignites and continues to burn as long as it is fed with wire. It is in this feeding" (it adds) "that the great difficulty has resided."

In the *Druggists' Circular* for January, 1866, we find announced, among the excerpts, the invention, in Edinburgh, of a lamp for burning magnesium, by which, it is stated, "all the difficulties of using this light for streets, public buildings, light-houses, and so on, are overcome." This lamp is described as being "of a character so simple and effective, that all the mechanicians are astonished. It is one of those happy ideas that seem inspired, and at the same time make everybody wonder they had not thought of them. The magnesium is reduced to a fine powder, then mixed with sand; it runs through a tube as from an hour-glass, and when lighted by a match, a brilliant and steady flame is produced until the reservoir is exhausted." The effect of the sand, as in this case, would be to moderate and equalize the combustion.

This, however, does not appear to have met the expectations raised in regard to it, certainly has not come into general use, and the lamps now employed are constructed for burning the metal in the form of ribbon, which is "paid out" by means of a clock-work arrangement. A recent improvement on this plan, is described by A. R. Leeds, A. M., Professor of Chemistry in the Philadelphia Dental College, in the *Dental Cosmos* for April, 1867, in an article on "The Magnesium Light," wherein the author, among other obvious uses, directs attention to the applicability of this light to Dental purposes, as in the illumination of the interior of the mouth, whether for mere inspection or operation. (And it is this "Odontoscopic" view of the subject that has prompted the present communication.)

But notwithstanding the devices of ingenuity, the great inherent difficulty remains, as to the rapid combustion of the metal, as well as the liability of the flame becoming extinguished, as it would seem, by its own violence. When used in the form of ribbon it burns away still more rapidly than in the form of wire, although the flame is more equable, less liable to extinguish itself, and freer from other objections.

This difficulty, however, may in a measure be removed, and some advantages obtained by burning the metal in *connection with zinc.* It is just about a year ago since I demonstrated this experimentally (on the 4th of July last). Perceiving the practical objection in the way of burning magnesium by itself for illumination, owing to its speedy combustion, etc., it occurred to me that the using of some other metal in connection with it, might be of advantage in controlling or modifying the light; and from the property of *zinc* to take on combustion at a bright red heat, I inferred that the heat of the ignited magnesium would be sufficient to produce and keep up the combustion of zinc, which in its reaction might serve not only to equalize, but also to perpetuate the combustion of the magnesium. Having, accordingly, hammered down a piece of magnesium wire (not having the metal in the form of ribbon at hand), and also a narrow strip of sheet zinc to about the same thickness, and pinning the two together with little zinc pins, I ignited the conjoined strip in the flame of a spirit lamp, when the whole burned steadily with a bright white light until completely consumed, but more slowly than magnesium by itself, leaving the "ashes" or oxides of both intimately cohering the entire length. A similar strip of zinc alone, ignited under the jet of a blow-pipe (though not in the ordinary spirit flame), burning with a greenish blue-white flame for a moment, but only for a moment, requiring to be rekindled each time; I intended to experiment a little more systematically, after procuring some magnesium ribbon of uniform thickness; but other matters called at that moment, (and time flies!) so it was not until reading Prof. Leed's interesting communication that I recurred to the subject.

On repeating the experiment with magnesium ribbon, thickness No. 36 of Brown & Sharps Standard Gauge, pinned to a strip of zinc rolled to No. 34, the piece burns slowly, and when held with the zinc side up is soon extinguished, but with the magnesium side held up burns steadily to the end. With strips of zinc No. 34 on both sides, it ignites with more difficulty and goes out after a moment's burning. but with thinner strips of zinc on each side, say about No. 38 (my gauge measuring only to 36), it burns steadily and completely, even better than with zinc No. 34 pinned to one side only. Edge up appears to be the best position for burning, when held horizontally. Using single strips of both metals of the same thickness (No. 36), I find the united

"flambeau" to burn from two and a half to three times as long as the magnesium by itself. The zinc alone, when uniformly *semi-oxidized at a red heat, just below the point of combustion*, burns after once ignited until consumed, with a yellowish-white, or sun-like light.

The ZINCO-MAGNESIUM light is of a clear, pure white, hardly distinguishable from the Magnesium light, not quite so intense, however, but milder and steadier. It would seem to possess advantages which, in connection with its greater economy, may render it quite as useful and more generally applicable for purposes of illumination.

ALBANY, N. Y., June, 1867.

